# A Clinically Relevant Mouse Model of Concussion Incorporating High Rotational Forces

**DOI:** 10.1089/neur.2024.0165

**Published:** 2025-02-17

**Authors:** Elizabeth M. Teasell, Emilie Potts, Nicole Geremia, Lihong Lu, Xiaoyun Xu, Haojie Mao, Arthur Brown

**Affiliations:** ^1^ Robarts Research Institute, Schulich School of Medicine and Dentistry, University of Western Ontario, London, Canada.; ^2^ Neuroscience program, Schulich School of Medicine and Dentistry, University of Western Ontario, London, Canada.; ^3^ Department of Biomedical Engineering, Schulich School of Medicine and Dentistry, University of Western Ontario, London, Canada.; ^4^ Department of Mechanical and Materials Engineering, Schulich School of Medicine and Dentistry, University of Western Ontario, London, Canada.; ^5^ Department of Anatomy and Cell Biology, Schulich School of Medicine and Dentistry, University of Western Ontario, London, Canada.

**Keywords:** closed-head impact animal model of concussion with high rotational forces, concussion, diffuse axonal injury, mild traumatic brain injury (mTBI), mouse model of concussion

## Abstract

Clinically relevant models of concussion are critical in understanding the pathophysiology of concussion and its long-term outcomes. To bridge the gap between preclinical and clinical research, animal models of concussion should be produced by mild traumatic brain injuries (mTBIs) that possess the same physical and biomechanical properties found in the mTBIs that cause concussion in humans. Specifically, to have good construct validity the mTBIs used in animal models of concussion should feature closed-head impacts with unrestrained head and body motion, resulting in peak angular velocities that approximate the human experience. We describe a mouse model of concussion using a cortical impactor to deliver closed-head mTBIs. Mice are placed on a break-away platform that allows free head and body movement during and after impact resulting in rapid head rotation. We assessed this model of concussion in over 100 mice carrying humanized versions of the genes encoding the amyloid precursor protein and tau. We found that this method consistently produced injuries with peak angular velocities in mice that, when scaled, approximated the average peak angular velocities reported in concussive football impacts. Face validity of this model of concussion was evaluated by histopathology and revealed that three impacts delivered 24 hours apart led to diffuse axonal injury, astrogliosis, and microglial activation one week after injury, particularly in white matter tracts aligned orthogonally to the axis of rotation. Persistent axonal degeneration was observed up to 6 months postinjury. This mouse model of concussion captures key biomechanical and pathological features of human concussions.

## Introduction

Concussion may be defined as a transient impairment in neurological function due to a mild traumatic brain injury (mTBI), typically caused by a blow to the head or body.^1^ There is an ongoing need for clinically relevant animal models of concussion. To achieve construct validity, preclinical models should employ mTBIs that generate biomechanical forces consistent with those in human concussion, particularly the rapid changes in angular velocity, which is a key contributor to concussions in humans.^2–4^ Indeed, angular velocity produces much of the brain strain-induced damage, while linear velocity does not.^5,6^ As human mTBIs typically involve closed-head impacts, with the head and body free to move during and after impact, construct validity is enhanced in preclinical models where the mTBI is delivered to a closed head (no skin incision or craniotomy) and permits unrestrained movement of the animal.

The most common pathophysiological finding in human concussion is the presence of diffuse axonal injury (DAI), which is often accompanied by astrogliosis, microglial activation, and pathological tau phosphorylation.^7,8^ The vector of the biomechanical forces generated by an injury dictates the pattern of DAI, with initial forces determining the most affected white matter tracts.^9^ Mechanical strain and strain rate at impact are associated with patterns of glial activation and axonal injury.^9^ Thus, models demonstrating DAI, astrocytosis, microglia activation, and phosphorylated tau without causing gross damage will possess greater face validity.

Additionally, the validity of mouse models of concussion may be improved by humanizing mice for key genes involved in concussion pathophysiology. In humans, tau and amyloid aggregation are thought to be important drivers of pathology after TBI and in neurodegenerative diseases more generally.^8,10,11^ Since murine tau and amyloid do not aggregate under pathological conditions as their human homologs do,^12,13^ the use of knockin mice carrying humanized versions of these genes^13,14^ better models these pathological features. We herein describe a novel preclinical model of concussion in transgenic mice using a cortical impactor to deliver a mTBI to mice positioned on a break-away platform allowing for unrestrained head and body rotation after impact. The injury generates peak angular velocities of the mouse’s head that approximate the peak angular velocities produced during human concussion. We show that three impacts delivered 24 hours apart lead to astrogliosis, microglial activation, and persistent DAI in tracts oriented orthogonal to the axis of rotation produced with impact.

## Methods

### Animals

All protocols for these experiments were approved by the University of Western Ontario Animal Care Committee in accordance with the policies established in the Guide to Care and Use of Experimental Animals prepared by the Canadian Council on Animal Care. Male and female homozygous humanized amyloid-beta (hAβ; B6N;129S-App*^tm.11Aduci^*) and humanized tau (h*MAPT* KI; Mapt*^tm1(MAPT)Tcs^*) transgenic mice were bred in-house for the experiments.^13,14^

### Model of concussion

**FIG. 1.**
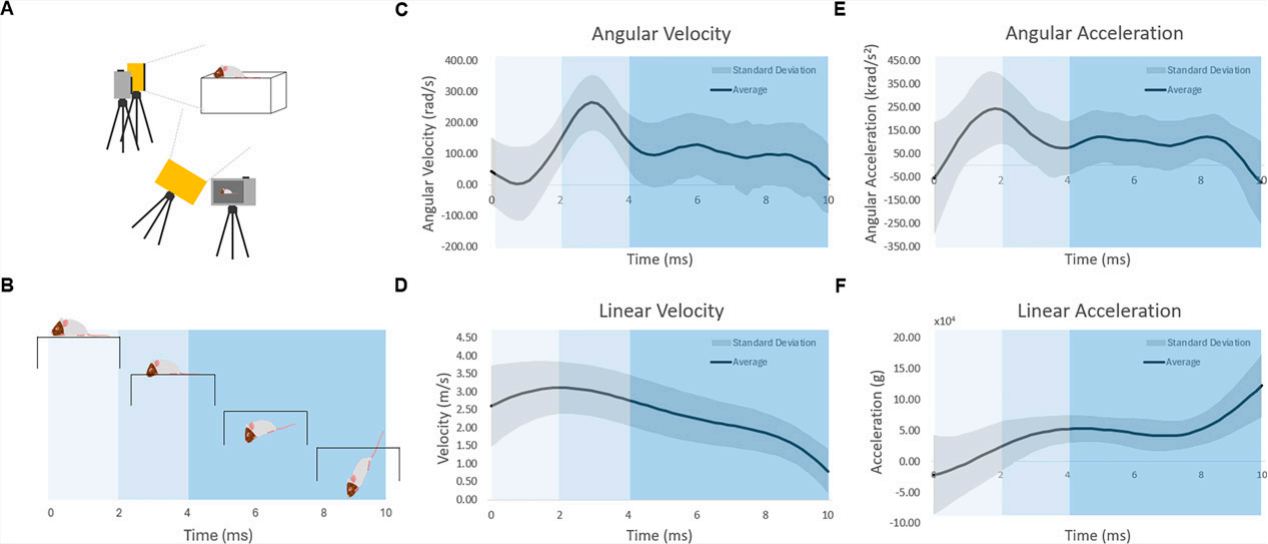
Head kinematics during rotational mTBI. Head kinematics were analyzed using head-speed videography during 297 impacts involving 102 mice (43 females and 59 males). **(A)** Cameras were positioned to capture both frontal and lateral views of each impact. **(B)** The time course of head and body movement during first 10 ms postimpact. The animal’s head breaks through the platform between 2 and 4 ms (light blue shading), followed by the body, which rotates 180° from its initial position to land on the back. Kinematic data **(C–F)** are presented as the mean ± standard deviation across all 297 impacts, with the peak angular velocity shifted to occur at the same time across all trials. mTBI, mild traumatic brain injury.

Adult (6 months old) male and female mice weighing 20–50 g were anesthetized by intraperitoneal injection with ketamine (80 mg/kg) and xylazine (10 mg/kg). The animal was placed on a break-away platform made of clear cellophane wrap (13-1300248, CADO, Montreal, QC) affixed to the top of a custom-made acrylic box (∼14 cm in depth) (Supplementary Fig. S1). To facilitate the fall, a 6 cm × 10 cm cross (+) was scored on the plastic, with an OLFA perforation cutter, equipped with a RB28 blade. The mouse was then positioned under a cortical impactor (TBI 0310, Precision Systems and Instrumentation, LLC, Lexington, KY) fitted with a custom-made, 4 mm diameter, pliant, silicone tip that was aligned with the bregma. The device was programmed to deliver an impact at a velocity of 3.5 m/s, an intended depth of 8.0 mm, and a 500-ms dwell time. The force of impact caused the mouse to break through the plastic with a forward rotation and land on a custom-made polyester-filled cushion (24 cm × 12 cm × 2.5 cm) at the bottom of the box (Fig. 1B and Supplementary Fig. S1). Sham animals received the same anesthesia without impact. For repetitive mTBI (rmTBI), mice received three impacts at 24-hour intervals.

### Video tracking

For video tracking of the mouse’s head during impact, a custom-designed nylon headcover with a void at the impact location and trackable dots was used. Impacts were recorded with two Chronos 2.1-HD (Kron Technologies, Burnaby, British Columbia) high-speed cameras to obtain front and lateral views at 640 × 480 resolution and a frame rate of 5406 frames/s. The testing field was illuminated with two small floodlights (Fig. 1A). Each impact video was analyzed via an open-source physics tracking software, Tracker Video Analysis and Modeling. Head movements were analyzed for 10 ms following impact from 56 data points for each tracker. Data were then imported into MATLAB (The MathWorks Inc., Natick, Massachusetts), and with the use of an in-lab code, both linear and angular properties were obtained.

### Tissue preparation, silver staining and immunohistochemistry

Male and female sham and injured mice (*n* = 4–5 per group) were collected at 1 week and 6 months following the last injury. Silver staining of 50 µm cryosections containing the corpus callosum and optic tract was performed using FD NeuroSilver kit (FD Neurotechnologies, PK301) as previously described.^15^ A semiquantitative analysis of the number of silver-stained fibers per section was carried out on 3–5 sections per animal per location (corpus callosum and optic tract; *n* = 4–5 per group) with each section given a score from 0 to 3 (0 = no silver-stained fibers; 1 = a few scattered silver-stained fibers; 2 = moderate staining; and 3 = strong staining). Immunohistochemical staining was carried out on 20 µm coronal cryosections incubated with primary antibodies: mouse anti-GFAP (1:1000, Millipore Sigma, MAB360) and rabbit anti-Iba1 (1:1000, Wako, 019-19741). Secondary antibodies used were Alexa Fluor 594-conjugated donkey anti-rabbit IgG (1:1000; Invitrogen, A21207) and Alexa Fluor 488-conjugated donkey–mouse IgG (1:1000; Invitrogen, A21202). Sections were mounted with VectaShield HardSet with DAPI (Vector laboratories, H-1500). Images were acquired on a DM6 B THUNDER imager at 20×.

## Results

### Injury kinematics

For each mTBI, the impactor was calibrated to deliver a 3.5 m/s impact at the bregma suture with a depth of 8.0 mm and a 500 ms dwell time. High-speed videography (set up shown in Fig. 1A, B) was used to calculate angular velocity and acceleration as well as linear velocity and acceleration (Fig. 1C–F). Across 297 impacts (102 mice), the average velocity was 3.56 ± 0.08 m/s (range: 3.25–3.91 m/s). Kinematic data were unavailable for nine impacts due to animal death or camera malfunction. As brain strain is primarily produced by rotational kinematics and angular velocity has been shown to correlate strongly with brain strain,^5,16,17^ we focused on analyzing the angular velocities produced by these injuries. The average peak angular velocity was 278.96 ± 87 rad/s (CV: 0.31) (Fig. 1C). Males had higher peak velocities (291.3 ± 94 rad/s) than females (261.17 ± 75 rad/s, *p* = 0.0011). Using an equal stress/equal velocity scaling factor of λ = (mass of human brain/mass of mouse brain)^1/3^ = 13.8,^18^ a velocity of 278.96 ± 87 rad/s in mice corresponds to a human-equivalent peak angular velocity of 20.2 rad/s, closely matching the 22 rad/s observed in human concussion.^19^

### Long-lasting DAI

**FIG. 2.**
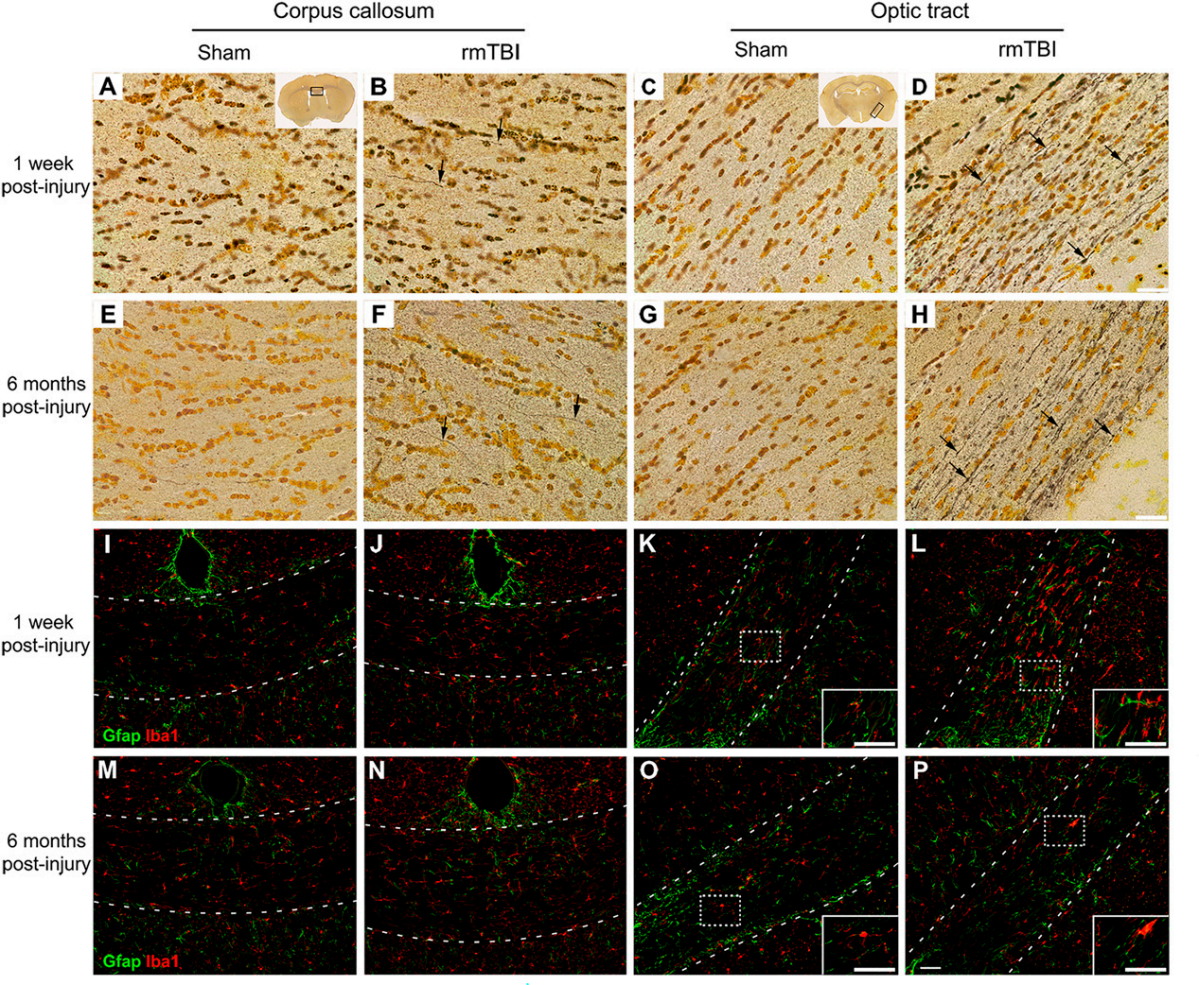
Repetitive mTBI induces diffuse axonal injury and microglia activation. Axonal injury was assessed with silver staining, and neuroinflammation was assessed by GFAP and Iba-1 immunofluorescence at 1 week and 6 months postinjury. **(A–D)** Silver staining in corpus callosum and optic tract at 1 week postinjury. **(E–H)** At 6 months postinjury, silver staining persisted in injured mice. Axonal swelling and beading are indicated by black arrows. Scale bar = 25 µm. **(I–P)** In the same white matter regions, microglia activation and astrogliosis were present in optic tracts, but not the corpus callosum, of injured mice at 1 week postinjury **(L)**. Scale bar = 50 µm. Representative images were chosen from each group with an *n* of 4–5 for both males and females for sham and mTBI groups at both time points. rmTBI, repetitive mild traumatic brain injury.

One of the initial and significant pathologies associated with mTBI is DAI. DAI disrupts axonal transport, resulting in the accumulation of degenerating cellular material, detectable by silver staining.^15,20,21^ To assess DAI, silver staining was performed 1 week and 6 months postinjury (Fig. 2). DAI was assessed in the corpus callosum and optic tracts, which primarily lie parallel and perpendicular to the axis of rotation, respectively. At 1 week postinjury, black silver-stained fibers with swellings and punctate beading (Fig. 2D, black arrows), characteristic of DAI,^15^ were evident throughout the injured brains. Staining was particularly evident in the optic tracts, with compartively less in the corpus callosum (Fig. 2B). The silver staining observed at 1 week post-mTBI persisted at 6 months postinjury (Fig. 2H). Scattered silver staining was also observed in the striatum, fornix, and internal/external capsule to varying degrees in injured mice. Few, if any, silver-stained fibers were seen in shams at either time point (Fig. 2A, C, E, G). A semiquantitative analysis of the number of silver-stained fibers per section failed to reveal any differences in the number of silver-stained fibers per section between male and female mice.

### The gliosis associated with axonal injury is temporary

At 1 week postinjury immunofluorescent staining for Iba-1 and GFAP revealed an increase in microglial and astrocytic activation in the optic tracts (Fig. 2L) but not the corpus callosum (Fig. 2J) of injured mice. Microglia in these tracts exhibited amoeboid morphology with few if any processes (inset Fig. 2L), characteristic of microglia primarily involved in phagocytosis.^22^ By contrast, the Iba1-positive microglia in sham mice were highly ramified and reminiscent of quiescent microglia (inset Fig. 2K vs. L). The activated glial response though pronounced at 1 week postinjury was greatly reduced by 6 months postinjury (Fig. 2P), although some mice exhibited amoeboid microglia and hypertrophic astrocytes in the optic tracts at this chronic timepoint. In contrast to the strong evidence for DAI produced by this model of concussion, there was no evidence of localized injury at the site of impact by gross inspection or by histological examination for neuronal loss or inflammation.

## Discussion

The present model of concussion is similar to previously described models that use weight drop impacts and either aluminum or Kimwipe break-away platforms.^23–25^ By combining a cortical impactor with a breakable transparent platform, the present method provides precise control over the mechanical impact and the potential for videography to measure the head kinematics associated with each impact.

Using this model, we recorded injuries in 102 mice, observing reliable and consistent kinematic signatures across impacts. The rapid head accelerations generated with this model produce peak angular velocities (278.96 ± 87 rad/s) that align with other established models, such as the CHIMERA (305.8 rad/s).^26^ Additionally, when scaled for human–mouse brain mass differences, the angular velocities achieved during our mTBI approximated the average peak angular velocity reported in football players undergoing concussive injuries.^19^

Rotational forces contribute to 90% of total brain strain during impacts, whereas linear acceleration contributes to a lesser degree.^16,27^ In this study, impacts were delivered to the top of the head, inducing a rotation about a right-to-left axis. Rotation about this axis would induce greater strain in tracts running in the anterior to posterior or superior to inferior orientations, but minimal strain in tracts parallel to the axis of rotation (right-to-left). In addition, preliminary simulations in the lab using a finite element mouse brain model incorporating fiber tracts showed minimal strain development for axons in the corpus callosum but more in the internal capsule.^28^ These strain rate predictions were supported by more abundant silver staining in optic tracts (anterior–posterior orientation) compared with the corpus callosum (right–left orientation). This supports the link between mechanical input and resulting functional damage.^29^

Chronic axonal injury observed at 6 months postinjury in our model is consistent with findings from other rotational injury models.^30–32^ This DAI persisted beyond that observed with a milder injury model we previously reported, where axonal degeneration was diminished at ten weeks postinjury.^15^ The greater persistence of DAI in the present model may be due to the larger rotational forces and driven by resulting secondary injury processes, including white matter-associated microgliosis and astrogliosis. The observed neuropathology is consistent with the long-lasting axonal degeneration and neuroinflammation documented in human TBI survivors.^33^

## Conclusion

The model of concussion presented has several key features: (1) free movement of the head and body at impact, (2) the transparent break-away platform facilitates high angular velocities and videography for kinematic analyses, (3) the cortical impactor allows for varied injury severities, and (4) the reported impactor settings produce mTBIs with peak angular velocities comparable to those experienced in human concussions. The injuries are highly reproducible, generate persistent DAI, microglial activation, and astrogliosis, in the absence of any gross structural damage. Thus, this model has strong construct and face validity with human concussion and may be ideal for laboratories investigating neurodegenerative processes after concussion.

## Transparency, Rigor, and Reproducibility Statement

The hAβ mice used in this study are commercially available from Jackson Laboratories (Jax Laboratories strain number 030898), while the humanized tau mice were acquired from Dr. Takashi Saito (RIKEN Center for Brain Science). h*MAPT* KI; hAβ double mutants were cross-bred to homozygosity in-house. Group sizes for the histological analyses were set to those generally employed in the field, with a sample size of five males and five females planned for each group. Littermates were randomized to sham or mTBI procedure. Experimenters were blinded to group identity. Antibodies used in experiments are well documented in the literature. The silver staining kit is well documented and commonly used in the field. Both 1 week and 6 months postinjury findings have been replicated by another member of the lab in different groups of mice. The injuries were produced using a cortical impactor that is commercially available from Precision Systems and Instrumentation. Materials and specifications to construct the break-a-way platform and box are fully described in this article. Videos of mice undergoing the injury were obtained over a 2-year period from August 31, 2021, to March 8, 2023. All injuries were performed between the hours of 8:00 am and 12:00 pm. Three impacts for each of the 102 mice were analyzed for kinematic calculations. Of the 102 mice, 2 mice died after the first impact, and 5 videos were lost due to camera malfunction or related issues. The coefficient of variation for the main kinematic measure, angular velocity, was 0.31. Figures, associated data sets, and videos from this article will be made available on Figshare. The full article data sets and figures will be freely available by contacting the corresponding author.

## Abbreviations Used

CV: coefficient of variation
DAI: diffuse axonal injury
GFAP: glial fibrillary acidic protein
hAβ: humanized amyloid beta
hMAPT: humanized tau
Iba-1: ionized calcium binding adaptor molecule 1
mTBI: mild traumatic brain injury
rmTBI: repetivie mild traumatic brain injury
TBI: traumatic brain injury
