# Supplementary material for: A Clinically Relevant Mouse Model of Concussion Incorporating High Rotational Forces
Source: Neurotrauma Rep. 2025 Feb 17;6(1):184–90. doi: 10.1089/neur.2024.0165 (PMC11931110; doi:10.1089/neur.2024.0165)
Supplement: Supplementary Figure S1 [file neur.2024.0165_supp_figs1.docx]

**
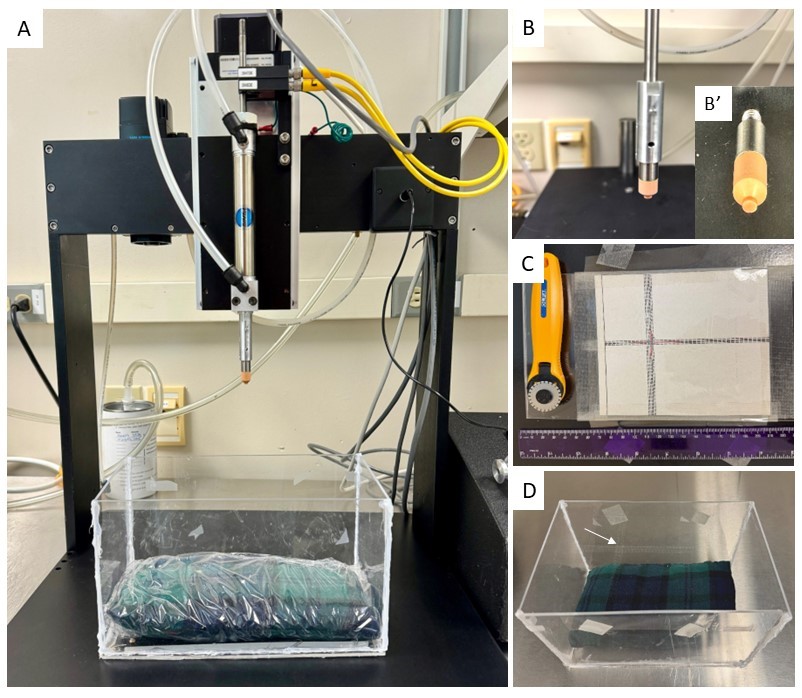
**

**Supplementary Figure 1.** **Set up for mTBI procedure.** (A) Full apparatus with cortical impactor positioned above box. (B) Piston and (B’) close up of custom-made, pliant 4 mm-diameter silicone impact tip (55 shore hardness) affixed over the standard 3 mm mouse metal tip with 2 mm silicone at impact surface and 0.5 mm on either side. (C) OLFA perforation cutter with RB28 blade and template used to score cellophane with 6 cm x 10 cm cross to facilitate the break through the platform. (D) Transparent acrylic box with cellophane platform and cushion in the base (11 cm below platform). White arrow points to scored plastic where the mouse is placed.
